# Mouse Models of Heterologous Flavivirus Immunity: A Role for Cross-Reactive T Cells

**DOI:** 10.3389/fimmu.2019.01045

**Published:** 2019-05-09

**Authors:** Mariah Hassert, James D. Brien, Amelia K. Pinto

**Affiliations:** Department of Molecular Microbiology and Immunology, Saint Louis University, St. Louis, MO, United States

**Keywords:** flavivirus, T cell cross-reactivity, heterologous immunity, original antigenic sin, Zika, dengue

## Abstract

Most of the world is at risk of being infected with a flavivirus such as dengue virus, West Nile virus, yellow fever virus, Japanese encephalitis virus, tick-borne encephalitis virus, and Zika virus, significantly impacting millions of lives. Importantly, many of these genetically similar viruses co-circulate within the same geographic regions, making it likely for individuals living in areas of high flavivirus endemicity to be infected with multiple flaviviruses during their lifetime. Following a flavivirus infection, a robust *virus-specific* T cell response is generated and the memory recall of this response has been demonstrated to provide long-lasting immunity, protecting against reinfection with the same pathogen. However, multiple studies have shown that this flavivirus specific T cell response can be cross-reactive and active during heterologous flavivirus infection, leading to the question: *How does immunity to one flavivirus shape immunity to the next, and how does this impact disease?* It has been proposed that in some cases unfavorable disease outcomes may be caused by lower avidity cross-reactive memory T cells generated during a primary flavivirus infection that preferentially expand during a secondary heterologous infection and function sub optimally against the new pathogen. While in other cases, these cross-reactive cells still have the potential to facilitate cross-protection. In this review, we focus on cross-reactive T cell responses to flaviviruses and the concepts and consequences of T cell cross-reactivity, with particular emphasis linking data generated using murine models to our new understanding of disease outcomes following heterologous flavivirus infection.

## Introduction

Both historically and currently, flaviviruses have had a huge global impact on human health. The four serotypes of dengue virus (DENV), yellow fever virus (YFV), West Nile virus (WNV), Japanese encephalitis virus (JEV), tick-borne encephalitis (TBEV), Murray Valley encephalitis virus (MVEV), and Zika virus (ZIKV) are among the most notorious members of this group. Globalization, climate changes, and vector overlap have caused more flaviviruses to co-circulate in the same geographic regions, increasing the likelihood that a person will be exposed to multiple flaviviruses throughout their lifetime. How exposure to multiple flaviviruses impacts the pathogen-specific immune response and alters the efficacy of flaviviral vaccines has been an area of intense research, combining both studies of human infections and animal models of heterologous flavivirus challenges. We know from decades of research that prior immunity to a flavivirus impacts the disease outcome (1–3). Considering this, and the established importance of the T cell response in disease outcomes of flavivirus infection (4), it is of the utmost importance to understand the effects of multiple flavivirus exposures on the development of protective immune responses against them. This review will provide an overview of the concepts and consequences of T cell cross-reactivity and what mouse models of flaviviral infection have told us and can teach us about heterologous T cell immunity.

Animal models of infection have provided important mechanistic insight into the role of T cells during flavivirus infection (5–13). Through the use of murine models, researchers have established a critical importance for T cells in protection from flaviviruses disease [reviewed in (4)]. The necessity of T cells is most clearly demonstrated through depletion or deletion studies, which show that in the absence of CD8+ T cells, uncontrolled viral replication and enhanced disease and mortality can occur in mouse models for JEV, ZIKV, WNV, YFV, and DENV infection (14–18). We have recently shown that a robust and polyfunctional CD4+ T cell response is elicited during ZIKV infection in mice (6). These cells infiltrate the CNS during infection, where they are able to restrict viral replication, resulting in limited disease and mortality (6). Importantly, they are absolutely critical for the prevention of ZIKV-induced paralysis in this model (6, 19). However, it is important to consider that, activities of antiviral T cells have also been reported to cause immune-mediated damage in the process of combating infection (20, 21). Paralysis and other neurological impairments seen in mouse models of neurotropic flaviviruses have been attributed to an aggressive neuroinvasive cytotoxic CD8+ T cell response in WNV, JEV, and more recently ZIKV (16, 19, 22). Due to the dual protective and immunopathogenic role of T cells during flavivirus infection, how the functional responses of these cells can change and impact disease outcomes in a heterologous infection environment requires further mechanistic investigation.

**Figure 1:**
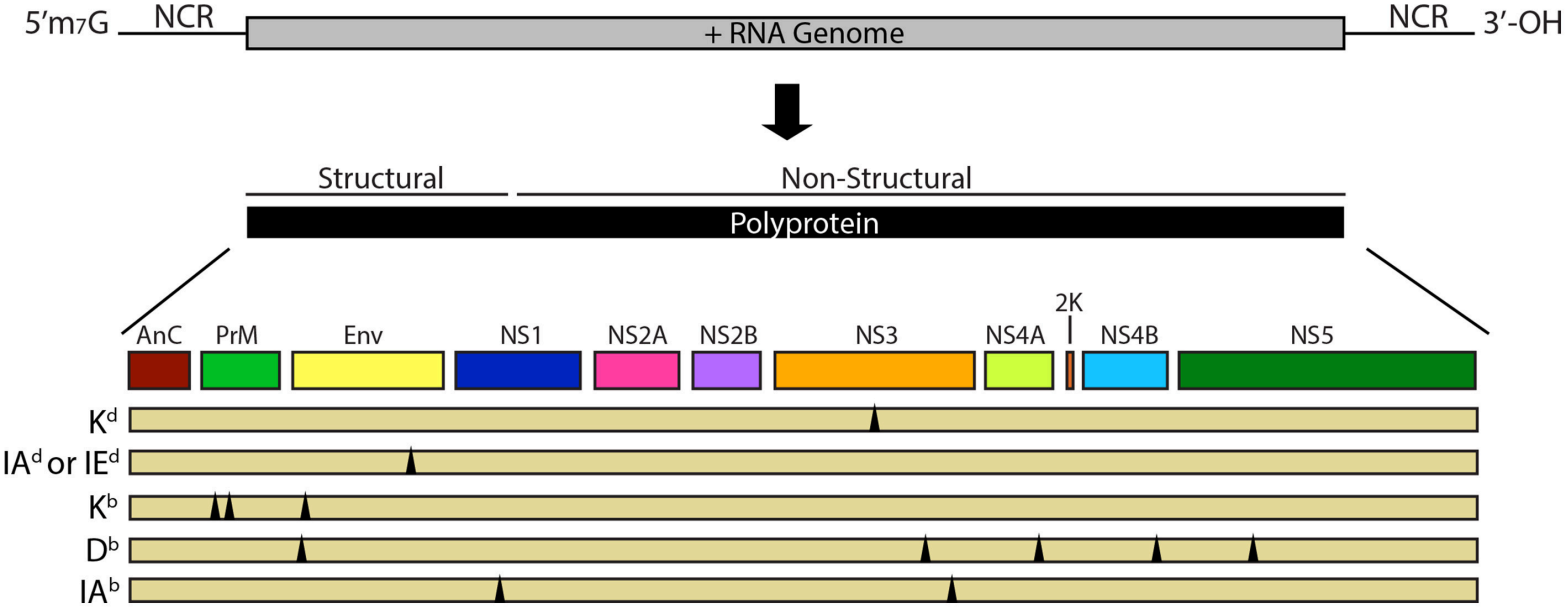
Flavivirus genome and proteins. The flavivirus genome consists of a single positive-stranded RNA molecule with a 5′ methylguanosine cap followed by an untranslated region (UTR), open reading frame (ORF) and a 3′ UTR with multiple variable stem loop structures. The genome is translated from a single ORF into a polyprotein that is proteolytically cleaved by both viral and host proteases. The genome codes for three structural proteins (capsid, membrane, and envelope) and seven non-structural proteins (NS1, NS2a, NS2b, NS3, NS4a, NS4b, and NS5). Theoretically, peptides of any of these structural or non-structural proteins have the potential to be targets of the virus-specific T cell response. Multiple flavivirus cross-reactive T cell epitopes with murine MHC restriction have been demonstrated in various murine models, the breadth of which are indicated by the triangles below the polyprotein. For detailed information on these identified cross-reactive epitopes see Table 2.

It is perhaps not surprising that T cell cross-reactivity exists between flaviviruses, as flaviviruses share between 30 and 70% amino acid identity across their coding region (23), and have a common genomic structure (Figure 1). The flavivirus genome is composed of a single positive stranded RNA, with a 5′ methylguanosine cap and a 3′ untranslated region with multiple variable stem loop structures (29). During replication, the genome is directly translated by host ribosomes into a single polyprotein that is subsequently cleaved by both viral and host proteases. Ten protein products are formed in total from these reactions including three structural proteins: capsid (C), membrane (prM/M), and envelope (E) and seven non-structural proteins: NS1, NS2A, NS2B, NS3, NS4A, NS4B, and NS5. All of these have the potential to be targets of the antigen-specific T cell response (29, 30).

The critical need to study immunological interactions that occur as a result of multiple flavivirus exposures is best exemplified by sequential DENV infection. DENV co-circulates in mosquito populations as four distinct serotypes. Immunity generated to one serotype does not confer protection against the heterologous serotype and instead, often results in enhanced disease (3, 31–33). How this phenomenon occurs is not fully understood, though there is evidence for both antibody-mediated and T cell-mediated mechanisms [reviewed in (20, 34)]. As we cannot control when we are exposed to pathogens in our lifetime, we have used animal models to address questions about how heterologous pathogen exposures shape the immune response and the consequences of T cell cross-reactivity. Murine models have provided an important tractable model for understanding the enhanced disease phenotype observed in heterologous DENV infection (35–37). More recently, these models are being adapted to explore the impact of prior flavivirus exposure on ZIKV immunity and pathogenesis (26, 38–41). Similar to the case of heterologous DENV immunity, cross-reactive T cell responses between DENV and ZIKV have become important to understand with the recent expansion of the geographic range of ZIKV infection (42–44).

## The Generation Of T Cell Cross-Reactivity

The T cell compartment is an arm of the adaptive immune system, which has the capacity to keep a record of past infections through immunological memory. Following T cell receptor (TCR) recognition of pathogen specific peptide epitopes presented on Major Histocompatability Complex (MHC) class I or II (45, 46) on antigen presenting cells (APCs), T cells expand and combat infection through various effector functions. However, of the considerable number of potential peptide sequences present during a given infection, only a relatively small fraction will be presented to and recognized by T cells to induce proliferation and effector function, which results in a numerical hierarchy of antigen-specific T cells termed “immunodominance” (47). With every immunological insult comes the potential for alterations to the T cell repertoire and the immunodominance hierarchy within the host.

### The Theoretical Necessity of TCR Cross-Reactivity

The enormous theoretical potential of the T cell repertoire is vastly larger than the number of T cells that can occupy a single mouse or human at a given time (10^8^ T cells in mice and up to 10^12^ in humans) (48, 49). It is also known that multiple T cells can express the same TCR, which can occur through homeostatic expansion of naïve T cells, proliferative maintenance of memory cells, and infection or vaccination-mediated boosting of T cells (50, 51). Taken together, calculations of TCR diversity have yielded estimates of 2 × 10^7^ TCRs in humans (49) and 2 × 10^6^ TCRs present in mice (52). Which presents a dilemma—in order to mount an adequate response to any theoretical pathogen, it would require the presence of many more T cell clones than are actually present in the body. It has been proposed that one way that the immune system deals with this is through TCR cross-reactivity (53). It has been calculated that given the maximal number of amino acid combinations for example an 11-mer peptide and controlling for restrictions in specific amino acids allowed at certain residues for presentation, that a given TCR may be capable of recognizing between 10^6^ and 10^8^ p-MHC ligands (53). Based on these calculations and experimental mouse and human data, we now know that T cell cross-reactivity between related and even un-related pathogens is not an extraordinary occurrence, but an inevitability.

As we now know that the specificity described by clonal selection theory, which suggests one TCR for every one peptide, is a mathematical impossibility, we can presume that TCRs are not strictly epitope specific. Experimentally, it has been shown that there is a level of promiscuity in peptide recognition (54–57) and that one TCR can recognize a number of different peptides, each having variable levels of amino acid homology. The ability of one TCR to recognize multiple peptide epitopes is the basis for T cell cross-reactivity [reviewed in (58, 59)]. T cell cross-reactivity can occur if T cells with TCRs primed against epitopes elicited during infection with one pathogen cross-react with peptide sequences presented during infection by a different pathogen [reviewed in (60)]. Because these memory cells are present at a higher frequency and lower activation threshold than naïve cells specific to the second pathogen, they have the potential to be preferentially “boosted” over responses to epitopes specific to the secondary pathogen.

### Original Antigenic Sin

**Figure 2:**
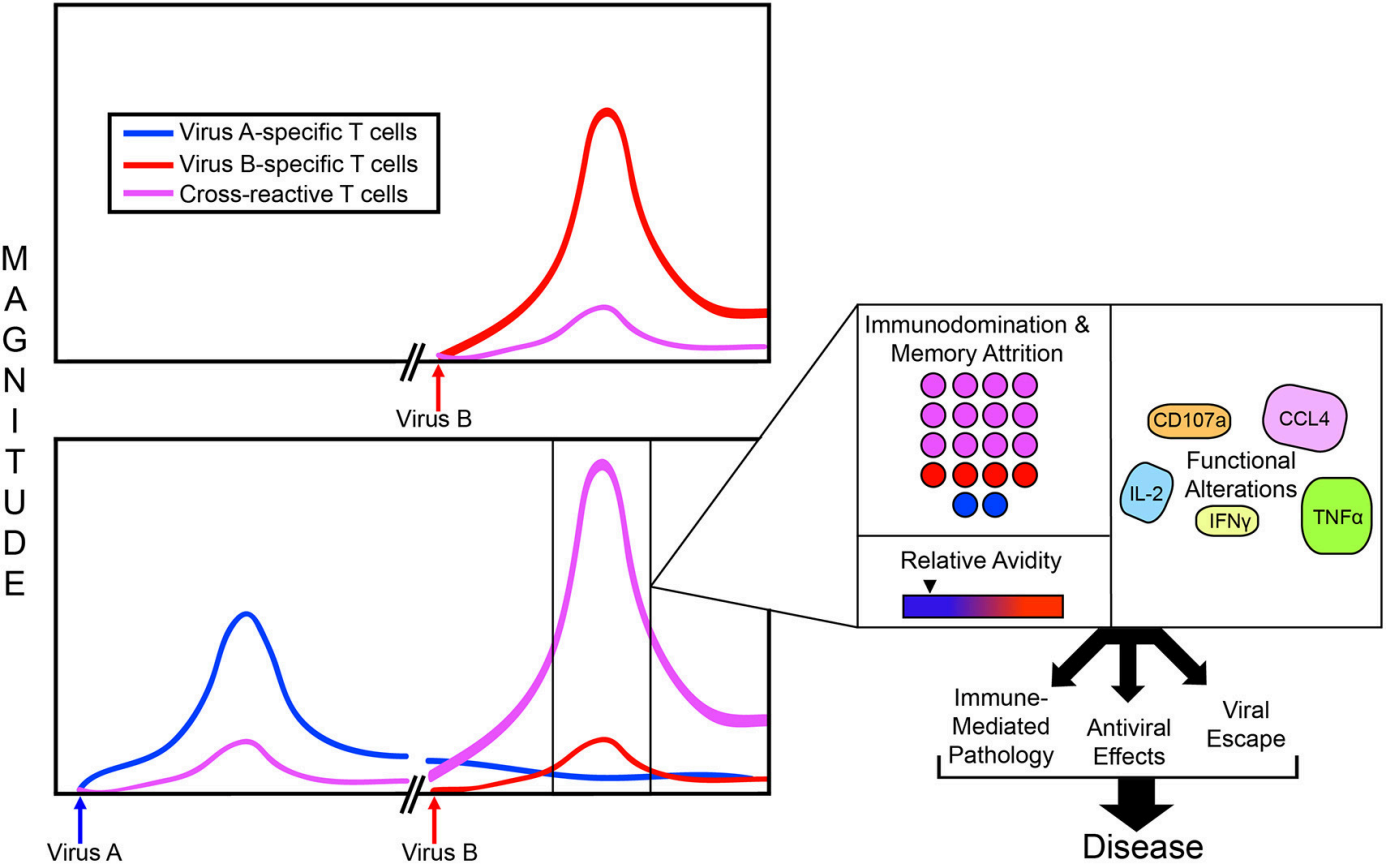
Consequences of T cell cross-reactivity during heterologous infection. During a primary infection, (for example with Virus B), a diverse T cell response may be generated against multiple Virus-B-specific epitopes (Red) possibly in addition to some cross-reactive epitopes (Purple); both of which will contract to some degree following viral clearance. However, if an infection with Virus B is preceded by Virus A, and the two viruses share responses to the same cross-reactive epitopes, an altered T cell immunodominance hierarchy may occur during the heterologous infection. In this case, at the point of infection with Virus B, cross-reactive memory T cells generated during infection with Virus A are already present at a higher frequency and lower activation threshold than naïve T cells specific for Virus A. This can lead to a preferential expansion of the cross-reactive T cells often at the expense of the virus specific ones, or “immunodomination.” During this process, memory cells specific to Virus A can even be lost from memory attrition, potentially impacting protection from future infections with Virus A. Sometimes, T cell cross-reactivity can occur in the absence of neutralizing antibody cross-reactivity, resulting in higher antigen loads than what would normally be present in a homologous boosted infection (Virus B followed by Virus B) which can lead to profound T cell activation of a higher magnitude. In the case of some flaviviruses cross-reactive antibody can even increase antigen load via ADE. The preferentially expanded, cross-reactive T cells can display different avidity compared to those that would have been generated during an infection with Virus A in the absence of prior heterologous exposure. During a primary infection with Virus A, the cross-reactive population would normally have a stronger avidity to the peptide variant of Virus A. However, during a heterologous infection, they have a stronger avidity to the peptide variant of the prior infection, Virus B. T cell cross-reactivity during heterologous infection can even have functional implications for cross-reactive T cells, though the alterations to cytokine profiles and their consequences is often virus-specific. All of these alterations to T cell populations and their functional capacities will dictate the balance between cross-protection and immunopathology, and can even result in viral escape; The sum of these, ultimately defining the disease outcome.

Each infection can induce lasting changes to the individual's T cell repertoire because a portion of the antigen-specific T cells generated in response to infection will be retained into memory. These antigen specific cells remain at a higher frequency and lower activation threshold, so that they can be recalled and mount a more rapid and effective response if the same antigen were to be encountered again. The presence of a highly functional antigen specific memory lymphocyte population at a higher frequency than that of the naïve population is the fundamental basis for vaccination (61). However, in instances of T cell cross-reactivity between two pathogens, during the secondary heterologous infection cross-reactive memory T cells can preferentially expand over more pathogen specific naïve ones precisely due to elevated frequencies and reduced threshold for activation, in a phenomenon termed “Original Antigenic Sin” (OAS) (Figure 2) (5, 62–66). As discussed in greater detail below, altered effector functions of these cross-reactive T cells that are primed to rapidly respond to cross-reactive antigens can have profound impacts on the balance between the protection and pathogenesis.

## Immune Response to Denv in Humans

Of the studies on cross-reactive immune interactions between flaviviruses, DENV has the longest history of investigation. The term “DENV” refers not to a single virus, but to a group of four (DENV1-4) genetically similar serotypes transmitted primarily by Aedes spp. mosquitos (67). Many DENV infections cause a range of symptoms from inapparent to mild, characterized by chills, fever, general malaise, retro orbital pain with presentation of leukocytopenia and thrombocytopenia, lasting for 4–7 days (68). However, in a small subset of patients, dengue hemorrhagic fever (DHF) will occur and is characterized by increased vascular permeability, loss of plasma volume and the characteristic “cytokine storm” which can lead to shock. The basis for this pathological progression is thought to be multifactorial; involving elements of host genetics and immunological background, as well as viral-intrinsic factors, though the immunological background of the patient has been shown to be one of the main predictors of disease outcome (69).

### Cross-Reactive Responses to DENV

Immunity to one serotype of DENV confers apparent lifelong protection from the same serotype and a brief period of heterotypic immunity to the other serotypes. However, following this window of cross-protective immunity, the cross-reactive adaptive immune response has the potential to enhance disease in infection with a heterologous serotype, increasing the risk of developing DHF by 15 to 80-fold (3, 69). One mechanism for enhanced dengue disease, first proposed by Halstead, is antibody-dependent enhancement (ADE) (34). It has been shown in *in vitro* and *in vivo* models that cross-reactive antibodies present at sub-neutralizing concentrations can promote DENV uptake into Fcγ-bearing cells leading to enhanced viral loads (37, 70–73). However, owing to the fact that DHF occurs *after* the peak of DENV viremia and closer to the peak in the T cell response, cross-reactive T cells have also been proposed to play a role in the pathology observed (20). It is important to consider that during a homologous secondary infection, the type-specific neutralizing antibody response functions to restrict the replication of virus, in effect lowering the antigenic load during T cell priming. Consequently, the boosted memory T cell response elicited may only be of modest size as this is dependent upon antigenic load. However, in a heterologous infection, the second infection may not be constrained by cross-reactive neutralizing antibody responses, and in the case of DENV, cross-reactive antibodies may even enhance the viral load (74). The large antigen load could drive a massive expansion of cross-reactive memory T cells, potentially leading to immune-mediated pathology, which is one hypothesis for the pathology observed during DHF (20).

In humans, DHF correlates with the magnitude of the T cell response and production of several cytokines, such as TNF-α, further providing a means for T cell cross-reactivity to play a role in disease severity (75). In addition to altered cytokine profiles during DHF, altered TCR avidities as a consequence prior DENV exposure have also been reported in humans. For example, in an analysis of a Thai cohort of DHF patients, it has been shown that the humans expressing HLA-A^*^11 possessed CD8+ T cells reactive to the NS3 epitope (NS3_133_) present in multiple DENV serotypes (75). While those T cells could bind tetramers containing peptide variants from multiple DENV serotypes, the avidity with which they did so varied based on the individual's serotype infection history, specifically with the lowest avidity attributed to the currently infecting serotype (76, 77). This observation supports the OAS hypothesis that cross-reactive cells of lower avidity are preserved in memory from a prior infection, then expand upon heterologous challenge, which yields T cell populations of lower avidity to the newly infecting serotype (76, 77). This was similarly demonstrated in an HLA-A^*^11 Vietnamese cohort of DENV-infected patients. In addition to these altered avidities, altered cytokine profiles in responses to the same cross-reactive variant peptide ligand as a consequence of secondary heterologous infection were also observed (78). In this case, the result of heterologous secondary infection was a skewing to the production of inflammatory cytokines TNF-α and CCL4 with decreased production of IFN-γ and IL-2 (78–80). This data supports the idea that T cell function can be impacted as a result of cross-reactive DENV infection in humans.

## Animal Models Of T Cell Cross-Reactivity

T cell cross-reactivity reshapes the pathogen specific T cell population. Exposure to a heterologous challenge alters the functional profile of a cross-reactive T cell relative to T cells that had not seen a heterologous challenge by: (1) altering functional avidity (27, 65, 76, 77), (2) skewing the immunodominance hierarchy (5, 62–66), (3) deviation of cytokine profiles (81–83), and (4) altering memory populations (64, 76, 84, 85). Cross-reactive T cells can drive the generation of viral escape mutants, which would not be observed in the absence of heterologous challenge (62, 86, 87). As T cell cross-reactivity can have a profound impact on protection and disease (20, 35, 36, 88, 89), it is critically important to understand how and when T cell cross-reactivity can occur and the implications of a cross-reactive T cell response.

### Lessons From Non-flaviviral Pathogens

Much of what we know about T cell cross-reactivity comes from the lymphocytic choriomeningitis virus (LCMV), with studies involving T cell cross-reactivity between flaviviruses coming to the forefront more recently. This has been eloquently shown in mouse models of T cell cross-reactivity between LCMV and Pichinde virus (PV). The immunodominance hierarchy of the T cell response to LCMV in C57BL/6 mice is predictable and stable, with most of the CD8^+^T cells targeting GP_33_, NP_396_, and GP_276_ with responses to the epitope NP_205_ being largely subdominant (90). Importantly, the NP_205_ subdominant epitope is cross-reactive in mice infected with PV (64). When mice are sequentially infected with PV followed by LCMV, the immunodominance hierarchy completely shifts and even alters the population of T cells preserved in memory (memory attrition). Specifically, the responses to the normally subdominant but cross-reactive NP_205_ dominate the T cell response in a heterologous challenge (64, 84) (Figure 2). This demonstrates that immunodominance patterns can be influenced by an individual's infection history.

In LCMV as well as other pathogens, cross-reactive T cells can display altered functional profiles during heterologous secondary infection, leading to altered pathogen control and potentially immune-mediate pathology. Functional avidity is thought to correlate with enhanced *in vivo* effector capacity (91). T cells with higher functional avidity achieve effector functions with a lower concentration of peptide and would therefore be thought to be better at controlling pathogens in low antigen environments. Functional avidity can be determined by exposing T cells to increasing concentrations of cognate peptide *ex vivo* and measuring changes in effector responses. As cross-reactive T cells are initially primed to a different peptide ligand, they can display suboptimal avidity for the new ligand during a heterologous infection (Figure 2) (20).

Alterations to T cell polyfunctionality have also been observed in some instances of heterologous immunity. One study investigating Epstein-Barr virus (EBV) and influenza A virus (IAV) cross-reactive T cell responses compared individuals experiencing mild vs. severe acute infectious mononucleosis (AIM). During EBV infection, patients with IAV-EBV cross-reactive T cell responses, had altered cytokine profiles and experienced severe AIM (92). As these studies were conducted with human volunteers, a distinct correlation between less severe disease and specific cytokine profiles could not be made, however this study did support much of the scientific concepts developed using murine models demonstrating that heterologous immunity can impact T cell functionality, which can play a key role in determining pathogenesis vs. protection.

It is now being appreciated that a more diverse T cell repertoire correlates with protection from viral infection, and it is thought that one reason for this involves a decreased likelihood of the generation of pathogen escape mutants (62, 93, 94). RNA viruses, including flaviviruses, have a highly error prone RNA polymerase that introduces mutations to the viral genome at a rate of ~1:10^4^ nucleotides (95). A consequence of a highly error prone RNA polymerase and a rapid replication rate is a virus with the potential to rapidly adapt to the constraints of immune restriction. In the context of heterologous immunity, focusing the T cell response to cross-reactive epitopes as opposed to promoting a highly diverse T cell repertoire drives the potential to select for viral mutants that escape this focused selection pressure (62, 86, 87). In the example above, heterologous infection with PV followed by LCMV results in extreme clonal dominance of the T cell repertoire to the cross-reactive epitope NP_205_. In a related study, the same authors found that this did indeed results in the generation of NP_205_ epitope escape variants (62), suggesting that alterations to viral intra-host population dynamics, as a result of skewed immunodominance hierarchies may be an important consequence of heterologous immunity that needs to be investigated further.

Predicting disease outcomes based on the occurrence of cross-reactivity between two pathogens, does not appear to be a “one-size fits all” interaction. The consequences of these complex interactions can culminate in enhanced disease in one combination of infections and cross-protection in another or even have no effect on overall disease outcomes (Table 1) [reviewed in (60)]. Again, using the example of LCMV, infection of LCMV immune mice with PV results in a 10X reduction in PV titers (88) which was shown to be mediated by cross-reactive T cells via adoptive transfer experiments. Similarly, it was shown that CD8+ T cell cross-reactivity occurs between LCMV and vaccinia virus (VV), and that CD8+ T cells from LCMV immune mice can provide protection from VV (88). However, in sequential challenge experiments with LCMV followed by VV infection, while VV was cleared much faster due to cross-reactive T cells, the mice suffered from IFN-γ mediated acute fatty necrosis as a result of cross-reactive immune-mediated pathology (88). This observation emphasizes the need to include context and thoroughly understand the mechanism of pathogenesis in specific viral infections to determine how immune cross-reactivity can walk the fine line between protection and immune-mediated pathology.

**Table 1:** Select references for examples of the impact of flavivirus T cell cross-reactivity and murine models of pathogenesis.

**Mouse strain**	**Priming virus**	**Challenging virus**	**Overall effect**	**Details of pathology**	**References**
AG129	DENV4	DENV2	Protective	Cross-reactive T cells mediate reduction in viral titers and enhance survival	(36)
AG129	DENV3	DENV2	Protective	Cross-reactive T cells mediate reduction in viral titers and enhance survival	(36)
AG129	DENV1	DENV2	Protective	T cells contribute to protection during heterologous infection but are not necessary nor sufficient for protection from mortality	(96)
Ifnar1^−/−^	DENV4	DENV2	Protective	Cross-reactive T cells mediate reduction in viral load and are required for reduction in morbidity	(36)
WT C57BL/6	DENV1	DENV2	Pathogenic	Elevated liver enzymes, low platelet counts, increased megakaryocytes in the spleen, more hematopoietic centers in the liver and increased vascular permeability. Observed phenotype requires TNF-α producing CD8+ T cells	(35)
WT C57BL/6	DENV2	DENV1	No effect		(35)
Ifnar1^−/−^	DENV2	ZIKV	Protective	Cross-reactive CD8+ T cells mediate some protection from ZIKV-induced morbidity and mortality	(26)
Ifnar1^−/−^ HLA-B*0702	DENV2	ZIKV	Protective	Enhanced viremia in mice deplete of CD8+ T cells during heterologous ZIKV challenge	(97)
Ifnar1^+/−^ pregnancy model	DENV2	ZIKV	Protective	Reduced fetal resorption and reduced viral burden	(38)

### DENV

Mouse models of DENV infection have been a valuable tool for answering specific questions regarding the potential contributions of T cells to pathogenesis and protection in heterologous infection [reviewed in (98)]. Some specific advantages to these models in studying this phenomenon include the conserved MHC haplotype and documented infection history in the laboratory setting, which allow for robust epitope mapping studies and reduced variability. WT C57BL/6 or BALB/c mice develop minimal DENV viremia and disease due to the restriction of replication by the type I IFN system, but still mount B and T cell responses that can be functionally evaluated in response to heterologous infection (24, 99). However, mice deficient in IFN-α/β or α/β/γ have been used to more closely mimic DENV disease (98). Depending upon the exact IFN deficiency, dose, route, age, and strain, these mice can display early viremia, elevated hematocrit, TNF-mediated plasma leakage, and elevated liver enzymes more similar to what is seen in severe DENV disease (37, 98, 100, 101).

Multiple strains of mice with various MHC restrictions have been used to study the consequences of flavivirus T cell cross-reactivity in heterologous infection on T cell populations and their functions (25, 35, 65, 102, 103). The cross-reactive epitopes identified in these studies are shown in Table 2 and Figure 1. In WT BALB/c mice, (H2^d^ restricted), cross-reactive CD8+ T cell responses to a peptide of the NS3 protein (NS3_298_) have been observed for the four DENV serotypes (102). In sequential challenge experiments in these mice, Rothman et al. observed that while the overall kinetics of the antigen-specific T cell response were similar in a primary vs. heterologous secondary challenge, a preferential expansion of responses to this cross-reactive NS3 epitope during secondary heterologous challenge was observed, leading to a shifted immunodominance hierarchy toward the cross-reactive epitope (65). Moreover, this boosted NS3 cross-reactive response was characterized by enhanced TNF-α production compared to primary infection, which is potentially significant given the link between TNF-α levels and disease severity in humans (104). Most mouse models of DENV pathogenesis utilize the C57BL/6 (H2^b^ restricted) background, warranting investigations of DENV T cell cross-reactivity within this MHC haplotype as well. On this background, cross-reactive CD8+ T cell responses have been described to be directed against a peptide of the NS4a protein (NS4a_249_) (25). Similar to cross-reactive CD8+ T cell responses seen in the WT BALB/c model of heterologous DENV infection, T cell responses to these H2^b^ restricted cross-reactive epitopes are preferentially boosted upon secondary challenge and drive a more TNF-α dominant cytokine phenotype (25, 35). These studies highlight the relevancy of the murine model to identify correlates of protection as well as the possible causative agents of immune mediated pathology.

Through genetic manipulation of MHC haplotype, mouse models can be used to understand heterologous DENV T cell responses in humans in a more controlled environment. Experiments with Ifnar1^−/−^ HLA B^*^0702 transgenic mice have been used in DENV sequential challenge and peptide vaccination experiments to understand the impact of DENV immune cross-reactivity in the context of human HLA (Table 3) (103). In one study, T cells from HLA B^*^0702 mice were infected with one of the four DENV serotypes, then stimulated with predicted peptide epitopes from both the homologous and heterologous DENV serotypes. Similar to studies completed using human PBMCs, the authors of this study observed alterations to cytokine profiles and functional avidity when the cells were stimulated with variant serotype peptides compared to the infecting serotype (103). Specifically, the cross-reactive cells displayed higher avidity to the peptide of the infecting serotype as opposed to the variant peptides, and when stimulated with a variant serotype peptide, the cells produced less IFN-γ, were less polyfunctional, and expressed less CD107a (a marker of cytotoxic degranulation). This intriguing study hints at possible differences in the effector functions of cross-reactive flavivirus specific T cell, and points to the need for further studies to explore the potential immune-pathologic or protective outcomes of T cells in the murine model.

**Table 2:** Flavivirus cross-reactive T cell epitopes with murine MHC restriction identified using various murine models, named in the same manner as the papers they were identified in.

**Epitope**	**Amino acid sequence**	**Viruses reported in**	**Mouse Strain**	**MHC restriction**	**References**
NS3_298_	ARGYISTRVGM ARGYISTRVEM	DENV1/3 DENV2/4	WT BALB/c	K^d^	(24)
NS4a_249_	YSQVNPLTL YSQVNPTTL	DENV1/3 DENV2/4	WT C57BL/6	D^b^	(25)
PrM_20_	ISFATTLGV LLFKTEDGV	ZIKV DENV	Ifnar1^−/−^	K^b^	(15, 26)
PrM_44_	ATMSYECPM DTITYKCPL	ZIKV DENV2	Ifnar1^−/−^	K^b^	(15, 26)
E_4_	IGVSNRDFV IGISNRDFV	ZIKV DENV2	Ifnar1^−/−^	D^b^	(15, 26)
E_7_	SNRDFVEGM SNRDFVEGV	ZIKV DENV2	Ifnar1^−/−^	K^b^	(15, 26)
NS3_347_	PSVRNGNEI PSIKAGNDI	ZIKV DENV2	Ifnar1^−/−^	D^b^	(15, 26)
NS5_18_	CAEAPNMKII ESEVPNLDII	ZIKV DENV2	Ifnar1^−/−^	D^b^	(15, 26)
NS4b_209_	GASSVWNATTAIGL GASAVWNSTTATGL	WNV JEV	C57BL/6	D^b^	(27)
NS1_132_	TFVVDGPETKECPT TFVVDGPETKECPD	WNV JEV	C57BL/6	IA^b^	(27)
NS3_563_	WCFDGPRTNTIL WCFDGPRTNAIL	WNV JEV	C57BL/6	IA^b^	(27)
E-pep_1_	SIGKAVHQVF	JEV WNV DENV	BALB/c	IA^d^ or IE^d^	(28)

**Table 3:** Flavivirus cross-reactive T cell epitopes identified using HLA transgenic murine models, named in the same manner as the papers they were identified in with the exception of (*)NS5_2695_.

**Epitope**	**Amino acid sequence**	**Viruses reported in**	**Mouse Strain**	**References**
NS3_1682_	LPAIVREAI LPSIVREAL	DENV2/1/3 DENV4	Ifnar1^−/−^ HLA-B*0702 transgenic	(103)
NS3_1700_	APTRVVAAEM APTRVVASEM	DENV2/3/4 DENV1	Ifnar1^−/−^ HLA-B*0702 transgenic	(103)
NS3_2070_	KPRWLDARI RPKWLDARV	DENV2 DENV3	Ifnar1^−/−^ HLA-B*0702 transgenic	(103)
NS4b_2280_	RPASAWTLYA HPASAWTLYA	DENV2/1/4 DENV1/3	Ifnar1^−/−^ HLA-B*0702 transgenic	(103)
NS5_2885_	TPRMCTREEF KPRLCTREEF	DENV2 DENV3	Ifnar1^−/−^ HLA-B*0702 transgenic	(103)
NS2a_75_	RPALLVSFIF	ZIKV DENV2	Ifnar1^−/−^ HLA-B*0702 transgenic	(97)
NS3_206_	APTRVVAAEM	ZIKV DENV2	Ifnar1^−/−^ HLA-B*0702 transgenic	(97)
NS3_574_	KPRWMDARV	ZIKV DENV2	Ifnar1^−/−^ HLA-B*0702 transgenic	(97)
*NS5_2695_	RPGAFCIKVL	ZIKV DENV2	Ifnar1^−/−^ HLA-B*0702 transgenic	(97)
NS5_539_	VPTGRTTW	ZIKV DENV2	Ifnar1^−/−^ HLA-B*0702 transgenic	(97)
Env P1	IRCIGVSNRDFVEGMSGGTW FNCLGMSNRDFLEGVSGATW AHCIGITDRDFIEGVHGGTW MRCVGIGNRDFVEGLSGATW MRCIGISNRDFVEGVSGGSW MRCVGVGNRDFVEGLSGATW MRCVGVGNRDFVEGVSGGAW	ZIKV WNV YFV DENV1 DENV2 DENV3 DENV4	AG129 HLA-DR1, DR15, DQ8 transgenic	(40)
Env P25	ALVEFKDAHAKRQTVVVLGS HLVEFEPPHAATIKVLALGN LLVTFKTAHAKKQEVVVLGS RMVTFKVPHAKRQDVTVLGS	ZIKV YFV DENV1 DENV4	AG129 HLA-DR15 transgenic	(40)
Env P41	HRSGSTIGKAFEATVRGAKR FKKGSSIGKMFEATARGARR YKKGSSIGKMFEATARGARR	ZIKV DENV1 DENV3	AG129 HLA-DR15 transgenic	(40)
Env P7	YEASISDMASDSRCPTQGEA IEAKISNTTTDSRCPTQGEA IEAKLTNTTTESRCPTQGEP IEGKITNITTDSRCPTQGEA IEALISNITTATRCPTQGEP	ZIKV DENV1 DENV2 DENV3 DENV4	AG129 HLA-DQ8 transgenic	(40)
Env P8	DSRCPTQGEAYLDKQSDTQY KAACPTMGEAHNDKRADPAF DSRCPTQGEATLVEEQDANF ESRCPTQGEPSLNEEQDKRF DSRCPTQGEAVLPEEQDQNY	ZIKV WNV DENV1 DENV2 DENV3	AG129 HLA-DQ8 transgenic	(40)

*In the original research publication the amino acid sequence was presumed to be ZIKV NS4b (NS4b_426_). However our sequence alignment studies show the peptide to be within the NS5 region and therefore the peptide was renamed in this review for the sake of clarity with the first amino acid being position 2695 of the polyprotein*.

Interestingly, when measuring disease outcomes as a result of DENV heterologous T cell responses, it appears that the genotype of the mouse, strain of virus used, and order of infection are all variables that dictate enhancement of disease vs. protection. In sequential challenge experiments of Ifnar1^−/−^ or AG129 mice (which are globally deficient in type 1 interferon receptor or type 1 and type 2 interferon receptor, respectively), cross-reactive CD8+ T cells appear to play an important role in cross-protection, with a potentially minor role for CD4+ T cells (96). These mice, (similar to C57BL/6) are H2^b^ restricted—an MHC restriction in which DENV CD8+ T cell cross-reactive responses have been described against NS4a_249_ (25). Sequential challenge experiments in both AG129 and Ifnar1^−/−^ mice show that prior exposure to a heterologous serotype of DENV confers cross-protection from DENV2 challenge (36, 96). Depletion of various immune subsets following primary DENV infection have shown that B cells, CD8+ and CD4+ T cells are all important for mediating protection during DENV challenge (105). Cross-reactive CD8+ T cells, however play a particularly important role in mediating cross-protection, where antibody-mediated protection dominates in protection from a homologous serotype challenge (96).

However, when similar experiments are done in WT C57BL/6 mice, cross-reactive CD8+ T cells have been reported to enhance immune-mediated pathology (35). As explained above, while IFNα/β sufficient mice only support transient replication of DENV and do not suffer from disease as measured by weight loss or mortality, it appears that these mice can still suffer from immune-mediated pathology driven by heterologous secondary infection. In a sequential heterologous challenge experiment, one group found that infection with DENV1 (PR/94 strain) followed with DENV2 (Tonga/74 strain) resulted in elevated liver enzymes, low platelet counts, increased megakaryocytes in the spleen, more hematopoietic centers in the liver, prolonged bleeding times, and increased vascular permeability (35). Interestingly, if the order of infections was reversed, or if different strains of virus were used, this enhancement phenomenon did not occur. In support of this observation, is the notion that pathology and protection induced by T cell cross-reactivity during heterologous infection are not always reciprocal, which has been shown at length in LCMV mouse models of T cell cross-reactivity (60). Nonetheless, the authors of this study were able to show through adoptive transfer experiments that TNF-α producing CD8+ T cells were crucial for this enhanced disease phenotype. This supports the idea that altered cytokine profiles as a result of T cell cross-reactivity (which have been observed extensively in human DENV infection) can drive immunopathology (25, 65, 78–80).

It is clear that while progress has been made in understanding the functional consequences of heterotypic DENV immunity on T cell expansion and function, we still have a long way to go in understanding the consequences for disease outcomes. Discrepancies in disease outcome during heterologous challenge between different genotypes of mice with the same MHC type may in fact be the unintentional result of the methods by which disease is measured in the two models. This represents a limitation with the animal models of DENV disease, which requires careful consideration when drawing conclusions from the data regarding heterologous immunity.

### DENV and ZIKV

Following the introduction of ZIKV to the Americas sometime in 2013, a devastating outbreak ensued. While generally ZIKV infection is asymptomatic in ~80% of infected adults, the spread of ZIKV throughout the Americas was followed by severe disease outcomes including congenital Zika syndrome (CZS) in some fetuses born to infected mothers, Guillain-Barrè syndrome (GBS), encephalitis, uveitis, and severe thrombocytopenia (106). One hypothesis put forth to explain these unique observations of ZIKV pathogenicity was that prior heterologous flavivirus exposure of individuals in the flavivirus endemic areas of South and Central America perpetuated ZIKV pathogenesis (1). Similar to the cross-reactivity between DENV serotypes, DENV and ZIKV cross-reactive antibodies have been reported in humans, non-human primates (NHPs), and mice, indicating a theoretical potential for ADE of ZIKV by pre-existing DENV immunity (5, 107, 108). Moreover, it has been observed in multiple studies in humans, NHPs, and mice that DENV immunity does modulate the cellular immune response to ZIKV—specifically that prior immunity to DENV leads to more robust CD8+ and CD4+ T cell responses during ZIKV infection (5, 43, 103, 109–111). These findings have provided a potential link between prior flavivirus exposure and ZIKV disease outcome. As we gather more epidemiological and experimental data using animal models, we are finding that the consequences of immune cross-reactivity between DENV and ZIKV may be different than that of immune cross-reactivity between the DENV serotypes. For example, in controlled NHP sequential challenge experiments, Pantoja et al. were unable to show that prior DENV immunity enhanced ZIKV disease (5). And longitudinal studies in a cohort of Nicaraguan children have even suggested a protective effect of existing DENV immunity on the outcome of ZIKV infection (2). As effective T cell responses remain a defined correlate of protection from flavivirus infection, the impact of prior flavivirus exposure on T cell responses during ZIKV infection is an area that has generated serious interest.

Mouse models of ZIKV infection have been extensively used to understand viral tropism, factors influencing neurological disease, transmission, adverse fetal outcomes, and the immune correlates of protection (6, 15, 112–119). However, due to its only recent re-emergence to the global spotlight, most of these ZIKV studies are done in immunologically naïve animals experiencing their first flavivirus infection. While this offers a deconvoluted analysis, it does not take into account immunological interactions that may take place as a result of immune cross-reactivity during a heterologous infection. It is clear that given the history of DENV and reports of ZIKV immune modulation by prior flavivirus exposure in humans and NHPs, that these studies are warranted.

Similar to murine models of ADE in heterologous DENV infection, ADE can be modeled in murine models of ZIKV infection through administration of specific subneutralizing concentrations of flavivirus cross-reactive antibody (120). However, it is unclear if this phenomenon can similarly enhance ZIKV disease in humans, particularly given the apparent protective effect of prior DENV exposure on ZIKV pathogenesis both in humans and NHPs (2, 5). It appears from the murine studies evaluating the impact of DENV immunity on ZIKV pathogenesis, that cross-reactive T cells play a protective role. In studies using Ifnar1^−/−^ mice cross-reactive CD8+ T cell epitopes have been identified between DENV2 and ZIKV that target both structural and non-structural epitopes (PrM_20_, PrM_44_, E_4_, E_7_, NS3_347_, and NS5_18_) (26). Responses to these cross-reactive epitopes are boosted during heterologous infection as measured by IFN-γ production in response to peptide stimulation (26). It was found that these cross-reactive cells contribute to protection from ZIKV in this model by adoptive transfer of CD8+ T cells from DENV immune mice into naïve mice prior to ZIKV challenge. It was reported that these cells have the potential to mediate reduced ZIKV viremia and moreover can mediate protection from ZIKV-induced fetal destruction in this established mouse model (26). While these are exciting developments, work still needs to be done to functionally characterize these cross-reactive T cell responses, how they can facilitate protection, and determine under what conditions, if any, these cells may drive more severe disease.

### Heterologous Immunity Between Other Flaviviruses

While much of the flavivirus cross-reactive T cell research focuses on the four serotypes of DENV as well as the newly emerged ZIKV, there have been studies addressing cross-reactivity between other flaviviruses. In humans, flavivirus cross-reactive T cell responses have been reported in both CD4+ and CD8+ T cells between DENV and WNV, DENV and YFV, and JEV and DENV, although functional studies have been limited (121, 122). Evidence of HLA-restricted T cell cross-reactivity between these other flaviviruses has also been corroborated using HLA transgenic mice (Table 3) (40). Some of the most exciting functional studies done to address cross-reactivity between other flaviviruses has been completed by Saron et al. who demonstrated that cross-reactive CD4+ T cell immunity to JEV altered the development of the immune response to DENV (105). In these studies, the authors demonstrated that a prior JEV infection specifically alter the follicular T cells leading to a change in the serocomplex immune response. Relative to DENV mouse models of heterologous T cell immunity, investigations into these cross-reactive responses are fewer in number, though an important contribution to the literature.

In WT BALB/c mice, vaccination with inactivated JEV results in cross-protection from lethal WNV challenge, whereas interestingly, the inverse challenge scenario only led to reduced disease severity, but equal mortality (123). The particular mechanism for this cross-protection remains unclear but, given that in this particular study the vaccine was an inactivated viral particle, it is likely that this cross-protection was mediated primarily through cross-reactive antibody. The specific contributions of JEV/WNV cross-reactive T cells have been more thoroughly investigated in H2^b^ restricted C57BL/6 mice (27, 28). Within this model a single cross-reactive CD8+ T cell epitope was identified as NS4b_209_ and two cross-reactive CD4+ T cell epitopes were identified as NS1_132_ and NS3_563_ (27). The authors of this study were able to investigate functional differences of these cross-reactive T cells and found that following vaccination with the live attenuated JEV-SA14-14-2, CD8+ T cells specific to the NS4b_209_ cross-reactive epitope perhaps unsurprisingly displayed a higher functional avidity to the JEV peptide rather than the WNV peptide variant (27). Interestingly, altered cytokine profiles were also observed, with CD8+ cross-reactive T cells from WNV infected mice being more polyfunctional than JEV-SA14-14-2 infected mice. While this provides an interesting lead in yet another potential incident of flavivirus cross-reactivity, further studies are needed to understand how these functional alterations could contribute to protection from disease.

## Conclusion

Exposure to a pathogen has the potential to shape the functional immune response to the next—with each infection leaving an *immunological signature* housed within the memory of the adaptive immune system. This signature has the potential to alter immune responses to subsequent homologous and heterologous pathogen exposures. For flaviviruses, cross-reactive T cells can both limit infection and disease (4) and cause substantial immune-mediated pathology (20). This apparent dichotomous role of cross-reactive T cells in protection and pathogenesis makes it crucial to understand what factors drive this delicate balance. Mouse models of heterologous infection have been used to study flavivirus cross-reactive T cell responses, providing strong mechanistic insight into the impact of both pathogenic and protective T cell cross-reactivity. Based on these studies, many laboratories, including ours, are looking toward the future and using animal models to define the main drivers of immune-mediated disease enhancement and cross-protection mediated by flavivirus cross-reactive T cells. We are using animal models to test the feasibility of pan-flavivirus vaccines and broadening our study of flavivirus cross-reactivity to include overlooked endemic flaviviruses including Rocio and Powassan. Within such a diverse pathogenic and immunogenic family of viruses, prior studies of flavivirus cross-reactivity provide a strong foundation to both understand fundamental concepts in immune mediated cross-reactivity and developed the next generation of flavivirus vaccines and therapeutics.

## Author Contributions

All authors listed have made a substantial, direct and intellectual contribution to the work, and approved it for publication.

### Conflict of Interest Statement

The authors declare that the research was conducted in the absence of any commercial or financial relationships that could be construed as a potential conflict of interest.
